# Competencia en cuidado espiritual en enfermería: Revisión integrativa de literatura*

**DOI:** 10.15649/cuidarte.2635

**Published:** 2023-09-03

**Authors:** Claudia Consuelo Torres-Contreras, Lina María Vargas-Escobar, Jorge Yecid Triana-Rodríguez, Wilson Cañon-Montañez

**Affiliations:** 1 . Universidad de la Salle, Bogotá-Colombia. Email: claudiaconsuelo@yahoo.com Universidad de la Salle Universidad de la Salle Bogotá Colombia claudiaconsuelo@yahoo.com; 2 . Universidad El Bosque. Bogotá-Colombia. E-mail: lmvargase@unbosque.edu.co Universidad El Bosque Universidad El Bosque Bogotá Colombia lmvargase@unbosque.edu.co; 3 . Universidad de la Salle, Bogotá-Colombia. Email: jytriana@unisalle.edu.co Universidad de la Salle Universidad de la Salle Bogotá Colombia jytriana@unisalle.edu.co; 4 . Universidad de Antioquia, Medellín, Colombia. Email: wilson.canon@udea.edu.co Universidad de Antioquia Universidad de Antioquia Medellín Colombia wilson.canon@udea.edu.co

**Keywords:** Espiritualidad, Enfermería Holística, Educación en Enfermería, Educación, Investigación en Enfermería, Spirituality, Holistic Nursing, Education, Nursing, Education, Nursing Research, Covid-19, Enfermería, Pandemias, Enfermeras Practicantes, Salud Laboral

## Abstract

**Introducción:**

El cuidado espiritual puede considerarse un elemento central de la filosofía del cuidado holístico.

**Objetivo:**

Identificar investigaciones de intervención con perspectivas y estrategias educativas para el desarrollo de competencias en cuidado espiritual.

**Materiales y métodos:**

Revisión de la literatura en el período 2001-2021 de las bases de datos PubMed, ProQuest, Scopus, Lilacs y BVS (Biblioteca Virtual en Salud). Se siguieron las recomendaciones PRISMA y se basaron en parámetros definidos por Whittemore y Knafl para la identificación de problemas, búsqueda bibliográfica, evaluación y análisis de datos.

**Resultados:**

se encontraron 10 artículos de intervención publicados. Los entrenamientos relacionados con el desarrollo de habilidades para el cuidado espiritual fueron realizados en programas de formación superior del personal de enfermería. Las estrategias de instrucción son cursos específicos, sesiones cortas o programas a lo largo del plan de estudios. Las intervenciones muestran efectos sobre las habilidades y conocimientos. La espiritualidad individual de los estudiantes predice la capacidad de brindar atención espiritual.

**Conclusiones:**

Hay evidencia de estrategias para desarrollar la competencia de estudiantes y profesionales de enfermería en cuidado espiritual, aún son escasas las publicaciones sobre el tema, por lo que se necesitan nuevas y más rigurosas estrategias para desarrollar competencia en este enfoque de la disciplina de enfermería

## Abstract (en)

**Introduction:**

Spiritual care can be considered a basic component of the philosophy of holistic care.

**Objective:**

Identify intervention studies with educational perspectives and strategies for developing spiritual care competencies in nurses and nursing students.

**Materials and methods:**

Integrative review of literature conducted in the period 2001-2021, in the databases PubMed, ProQuest, Scopus, LILACS, and VHL (Virtual Health Library). PRISMA statement guidelines were followed for the review, and it was based on Whittemore and Knafl's stages for integrative reviews: problem formulation, literature search, evaluation of data, data analysis, and presentation of results.

**Results:**

Ten published intervention articles were found. Education for the development of spiritual care competencies has been provided by educational programs and activities in undergraduate nursing programs and with professional nurses in practice. Instructional strategies include specific courses, short sessions, or programs throughout the curriculum. Interventions have an impact on skills and knowledge. Students' individual spirituality is a predictor of the competence to provide spiritual care.

**Conclusions:**

There is evidence of strategies to develop spiritual care competencies in nursing students and professionals, but publications on the subject are scarce. Therefore, implementing new and more rigorous strategies that allow the development of competencies for approaching spirituality in nursing is required.

## Resumo

**Introdujo:**

o cuidado espiritual pode ser considerado um componente básico da filosofia do cuidado holístico.

**Objetivo:**

determinar estudos de intervengo com perspectivas e estratégias educativas para o desenvolvimento da competencia em cuidado espiritual em profissionais e estudantes de enfermagem.

**Materiais e Métodos:**

Revisao integrativa da literatura realizada no período de 2001-2021, nas bases de dados PubMed, ProQuest, Scopus, Lilacs e BVS (Biblioteca Virtual em Saúde). As recomendares do PRISMA foram seguidas e baseadas nos parámetros estabelecidos por Whittemore e Knafl para identificado do problema, busca na literatura, avaliagáo dos dados, análise dos dados e apresentagáo dos resultados.

**Resultados:**

foram encontrados 10 artigos de intervengo publicados. A educado para o desenvolvimento de competencias no cuidado espiritual tem sido realizada a partir de programas e atividades educativas, na graduado e nos profissionais de enfermagem durante sua prática. As estratégias de ensino sáo cursos específicos, sessóes curtas ou programas ao longo do currículo. As intervengóes mostram impactos nas habilidades e competencias de conhecimento. A espiritualidade individual nos alunos é um preditor da capacidade de fornecer cuidados espirituais.

**Conclusóes:**

Existem evidencias de estratégias para desenvolver competencias no cuidado espiritual em enfermagem em estudantes e profissionais, as publicagóes sobre o assunto sáo escassas, pelo que é necessária a implementagáo de estratégias inovadoras e mais rigorosas que permitam o desenvolvimento de competencias para a abordagem em Enfermagem.

## Introducción

Un aspecto fundamental de la enfermería es ayudar a las personas a proteger, mantener o lograr la salud física, psicológica, social y espiritual. De esta forma, el acercamiento espiritual del equipo de salud a los pacientes y sus familias en situaciones de enfermedad, vulnerabilidad o riesgo de muerte es una necesidad expresa del paciente^1^.

Por lo tanto, el cuidado espiritual puede considerarse un componente central de una filosofía de cuidado holístico^2^ y puede definirse como el cuidado de la conciencia y la respuesta del espíritu humano frente a eventos que cambian la vida (p. ej., nacimiento, trauma, accidente). o tristeza), incluida la necesidad de significado, autoestima, autoexpresión, apoyo de fe, tal vez ritual, oración o sacramento, o simplemente un oyente sensible^3^.

El cuidado espiritual comienza con el fomento del contacto humano en una relación compasiva que mejora la dimensión espiritual de la salud del paciente y aumenta la posibilidad de mejorar la recuperación al promover la esperanza y la participación en el tratamiento^1^. Las enfermeras deben conocer las creencias y prácticas espirituales de sus pacientes, y deben estar preparadas para ofrecerles atención espiritual personalizada. Se ha demostrado que las enfermeras no han recibido educación en cuidado espiritual durante su formación; sin embargo, reconocen la importancia de abordar este cuidado en sus pacientes como parte de una atención integral y holística^4^^-^^6^.

En la actualidad se han desarrollado diferentes propuestas desde enfermería para brindar educación a profesionales y estudiantes de enfermería que permitan disminuir esta barrera en la práctica, la mayoría en países europeos, Asia y USA donde se han elaborado las competencias para que los profesionales de enfermería fortalezcan los conocimientos, habilidades y prácticas en cuidado espiritual, como también se han generado estrategias de entrenamiento en esta temática^3^^,^^7^^-^^10^.

El objetivo de este artículo es determinar los estudios de intervención educativa para el desarrollo de las competencias en cuidados espirituales en profesionales y estudiantes de enfermería, que se encuentran disponibles en la evidencia científica. Focalizando en estudios empíricos en el ámbito de la educación en enfermería que permitan visualizar los desarrollos en la formación de la competencia en cuidados espirituales, investigando las intervenciones educativas y estrategias pedagógicas implementadas en la formación de profesionales de enfermería y enfermeros en este ámbito específico.

La competencia en cuidado espiritual se define como las capacidades en términos de conocimientos actitudes y habilidades del profesional de salud para evaluar y atender las necesidades espirituales de un paciente^1^^,^^11^^,^^12^ y es importante debido a que al tiempo que refuerza la resiliencia, desarrolla la capacidad de estudiantes y profesionales de Enfermería para proporcionar cuidados espirituales de personas y familias en diferentes ámbitos de la atención^13^.

## Materiales y Métodos

### Diseño y Registro del Protocolo

Revisión integrativa de la literatura que siguió las recomendaciones de PRISMA extensión for scoping reviews (PRISMA-ScR)^14^. El protocolo fue registrado en la plataforma INPLASY con el código 2021110081^15^, adicionalmente una muestra del análisis de calidad realizado a los estudios incluidos fue almacenada en el Mendeley Data: https://doi.org/10.17632/5f747ygsyd.1. ^16^

### Referente teórico de la revisión integrativa

La revisión se fundamentó en los criterios de Whittemore y Knafl^17^ que cumple con el rigor metodológico, a través del siguiente proceso: determinación del problema, pesquisa de literatura, valoración de la calidad de los datos, análisis y presentación de resultados.

### Fuente de los datos y estrategia de búsqueda

La información se obtuvo de las siguientes bases de datos: Medline (vía PubMed), ProQuest (vía EBSCO), Scopus, Lilacs y BDENF (vía Biblioteca Virtual en Salud - BVS). Las búsquedas fueron realizadas en los últimos 20 años hasta junio de 2021. En la Tabla 1 se puede observar en la estrategia de búsqueda en las bases de datos electrónicas.

**Tabla 1:** Estrategia de búsqueda para las bases de datos electrónicas.

PubMed/Medline	Scopus	ProQuest	Lilacs y BDENF
1.Spirituality	1.Nursing Education Research	1.Educations, Nursing	1.Nursing Education Research
2.Holistic Nursing	2.Spirituality AND Nursing	2.Nursing Educations	2.Holistic Nursing OR education
3.Spirituality AND Nursing	3.Spirituality AND Education	3.Spirituality	3.Holistic Nursing AND education
4.Spirituality OR Nursing	4.Spirituality OR Nursing		4.Spirituality
5.Spirituality OR Education	5.Spirituality OR Education		
6.Spirituality AND Education	6.Holistic Nursing AND education		
7.Holistic Nursing AND education	7.Holistic Nursing OR education		
8.Holistic Nursing OR education	8.Research’s, Nursing Education		
9.Nursing Education Research			
10.Research’s, Nursing Education			

### Criterios de elegibilidad de los estudios

Los artículos que respondieron a la pregunta de investigación incluyeron: Estudios de intervención en educación de enfermería espiritual para enfermeras o estudiantes de enfermería publicados y diseñados entre 2001 y 2021, texto completo disponible. Estudios de intervención (ensayos clínicos aleatorizados y estudios cuasiexperimentales) con o sin un grupo de control utilizando estrategias de cuidado espiritual o educación espiritual entre estudiantes de enfermería, enfermeras profesionales y estudios publicados en inglés, español y portugués. Se excluyeron los artículos que no establecieran claramente intervenciones o estrategias educativas o que fueran de campos no relacionados con la enfermería. Además, se realizó una búsqueda secundaria en las listas de referencias de los artículos encontrados. La Tabla 2 muestra las estrategias PICO revisadas.

**Tabla 2:** Descripción de la estrategia PICO

Definición	Descripción
P - Población	Profesionales de enfermería y estudiantes de enfermería
I - Intervención	Intervenciones educativas en cuidado espiritual o fortalecimiento de la espiritualidad
C - Comparador	Con o sin grupo control o cuidado estándar
O - (Outcomes - Resultados)	Competencias y habilidades para el cuidado espiritual

### Selección y recolección de la información

Se eliminaron los artículos duplicados después de realizada la búsqueda y los que no cumplían los criterios de inclusión. Luego, los autores prepararon una ficha de análisis que incluía los siguientes aspectos: autor, año, país, estrategias pedagógicas utilizadas para la intervención, desarrollo de conferencias y elementos conceptuales relacionados con la espiritualidad y la pastoral. Se realizaron dos lecturas independientes de cada artículo para valorar críticamente la evidencia; se cumplieron los criterios de inclusión y, posteriormente, los autores seleccionaron por unanimidad los manuscritos incluidos en la revisión. Esta incluyó artículos que cumplieron con los criterios de inclusión, artículos seleccionados que demuestran el impacto de una intervención educativa de enfermería en cuidado espiritual y que contenían elementos conceptuales y teóricos relevantes para la formación de capacidades para estudiantes y profesionales de enfermería.

### Análisis y presentación de la información

Para el análisis de la información inicialmente se hizo la caracterización de los estudios incluidos, posteriormente se realizó consenso de los autores para analizar los criterios de cumplimiento de cada artículo y finalmente se hizo un análisis de la información según cada pregunta, dando un panorama sobre el efecto de las intervenciones educativas enfocadas a la competencia de cuidado espiritual en enfermería.

Para minimizar los sesgos se hizo una búsqueda minuciosa con el fin de abarcar el fenómeno de estudio con información veraz y precisa.

### Evaluación de la calidad metodológica de los estudios

Se utilizó un enfoque analítico sistemático para evaluar los datos, lo que permitió la interpretación de datos de fuentes primarias, y los autores participaron de forma independiente en esta revisión, que luego se organizó de acuerdo a las intervenciones y modelo conceptual utilizado. Para evaluar la calidad de la evidencia científica analizada se utilizó el Qualification Criteria Tool for the Selection of Research Articles (ICrESAI)^18^, el cual consta de cinco dimensiones y nueve elementos que permiten determinar la selección de artículos, el mérito científico y la calidad metodológica para artículos que pueden ser incluidos en una revisión sistemática.

## Resultados

### Selección y caracterización de los estudios

**Figura 1:**
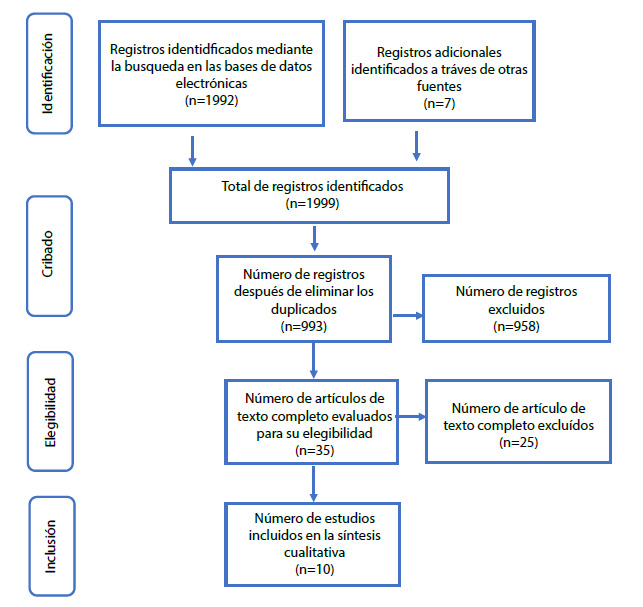
Flujograma para la selección e inclusión de los estudios

Se encontraron 1992 manuscritos en la búsqueda inicial y 7 artículos en la búsqueda secundaria. Después de refinar la búsqueda utilizando artículos que contenían alguna intervención educativa y/o de aprendizaje, se obtuvieron 993 artículos, luego de lo cual se aplicaron criterios de inclusión y exclusión. Se evaluó la calidad de la evidencia de 35 artículos, por lo que se incluyeron 10 artículos (Figura 1). Los artículos que fueron excluidos no contenían elementos conceptuales y teóricos del cuidado espiritual o porque no eran de acceso abierto y no evaluaban o comparaban claramente los efectos de la intervención.

*Fuente: Adaptado de la declaración PRISMA para publicación de revisiones sistemáticas y meta análisis*

Desde el punto de vista metodológico, un porcentaje importante de los estudios usan un diseño de tipo cuasiexperimental sin grupo control y con muestras seleccionadas a conveniencia, con participación voluntaria lo que pudiera emplear un sesgo de selección en los resultados^19^^-^^22^. Otros estudios (n=5) presentan aleatorización con grupo control lo que indica resultados más sólidos respecto al efecto^23^^-^^26^. Por fim, alguns participantes refletiram e reconheceram suas próprias vulnerabilidades, a despeito do imaginário de heroísmo que se construiu socialmente em torno da figura do trabalhador de saúde da linha de frente:

**Figura 2:**
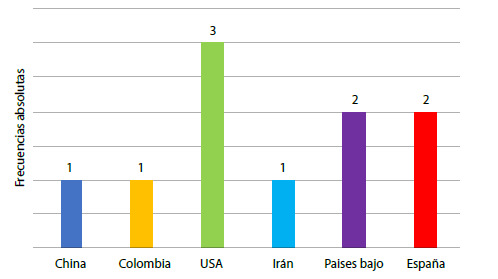
Descripciones de estudios de intervención en la competencia de cuidado espiritual según país periodo 2001- 2021

En la Figura 2 se observa que dentro de este grupo de publicaciones se han desarrollado principalmente en los países de Europa y USA, resaltando que solo uno en estudio en Colombia. Lo que permite ver un vacío en la formulación y uso estrategias educativas y/o pedagógicas claras y efectivas para el fortalecimiento de la competencia de cuidado espiritual en los profesionales y estudiantes de enfermería de Colombia.

### Intervenciones o estrategias educativas

La Tabla 3 muestra las características de las publicaciones que incluyeron componentes de la intervención de educación/enseñanza del cuidado espiritual que llevaron a la identificación de elementos pedagógicos para desarrollar estrategias y fortalecer la competencia en cuidado espiritual incluyendo: Modelos de Acción Espiritualidad y Cuidado Espiritual (ASSET) que incluyen: estructura y contenido, proceso y resultado, autoaprendizaje y práctica reflexiva. Se enfatiza en el uso de métodos pedagógicos como la reflexión individual y grupal, el trabajo en grupo, la expresión de experiencias y experiencias a través del diálogo grupal, el uso de guías de aprendizaje, el estudio de casos y el análisis a través del proceso de atención.

En la misma tabla se observan los estudios de investigación con efecto en los aspectos de educación, relacionados con la atención espiritual en la competencia de enfermeros y estudiantes de enfermería. Estos estudios reconocen diferentes tipos de programas educativos, algunos de ellos refieren la inclusión de la espiritualidad y el cuidado espiritual en los planes de estudio de los programas de enfermería, así como el impacto que han tenido en los estudiantes; otros se centran en la educación espiritual de grupos específicos de profesionales de enfermería. Las estrategias pedagógicas y los contenidos difieren en las formas de ofrecer los cursos, algunos se desarrollan a lo largo del plan de estudios 27 y otros en cursos específicos, así como tres estudios que presentan intervenciones con una o dos sesiones educativas únicamente^24^^,^^19^^,^^28^.

Lo que es común es encontrar en los artículos es el impacto de la educción sobre la competencia en el cuidado espiritual en enfermería, donde estudiantes y profesionales desarrollan más conocimientos acerca de la espiritualidad y mayores actitudes sobre la atención espiritual. También se demuestran mayores habilidades de comunicación y los participantes manifiestan algún grado de impacto personal. Sobre la base de estos estudios, se puede decir que la formación en cuidado espiritual tiene un efecto positivo en la competencia de las enfermeras.

### Efectos de las intervenciones en cuidado espiritual

En relación con los efectos de las intervenciones en cuidado espiritual desde el componente educativo, se evidencia un impacto en la competencia del profesional con importantes desarrollos en conocimientos y habilidades para la atención espiritual. La espiritualidad personal de los estudiantes es un predictor fuerte de la capacidad para proporcionar cuidados espirituales a pacientes y familias. Entre las herramientas de medición empleados en los artículos se resaltan: la escala de competencia de atención espiritual, el cuestionario de formación espiritual de los estudiantes de enfermería, la Escala de valoración de la espiritualidad y los cuidados espirituales, la Escala de salud espiritual, las Perspectivas de las enfermeras sobre la prestación de cuidados espirituales, la Autopercepción de la espiritualidad, el cuestionario de prestación de cuidados espirituales adaptado, el registro de necesidades espirituales y el cuestionario de satisfacción laboral, la Escala de experiencia espiritual, la Escala de Perspectiva del Cuidado Espiritual-Revisada, la Escala de empatía, la de Comunicación para la atención espiritual, Inventario de atención espiritual, la escala de la atención espiritual en la práctica, y la escala de bienestar espiritual. En esta se observan los cambios en las puntuaciones medias de cada una de las escalas demostrando la mejora en la dimensión espiritual en el grupo de intervención en la mayoría de los estudios incluidos en la revisión(Tabla 4).

**Tabla 3:** Publicaciones incluidas con componente de intervención educativa/pedagógica en cuidado espiritual 2001-2021

	Estrategia pedagógica- método Educativo	Tiempo de Desarrollo de la intervención	País	Población	Autores- Año	Elementos Conceptuales del cuidado espiritual
1	Guías didácticas- Estudios de Casos con Proceso de atención de enfermería^19^	2 sesiones	España	Estudiantes de enfermería	Fernández-Pascual et al., 2020	Espiritualidad intrapersonal.
						Espiritualidad interpersonal.
						Cuidado espiritual. cuidado espiritual. (introspección y autoconciencia)
2	Magistral (Video, ayuda didáctica y conferencia) ^24^	1 sesión	Colombia	Estudiantes de enfermería	Vargas-Escobar and Guarnizo- Tole., 2020	Concepto de espiritualidad
						Cuidado espiritual
						Atención individualizada
						Religiosidad
						(Conceptualización de la espiritualidad y el cuidado espiritual de McSherry)
3	Conferencias discusiones de caso y grupos de asesoramiento.^29^	2 semanas cada 6 meses	China	Profesionales de enfermería	Hu et al., 2019	Educación espiritual, incluida la educación sobre la vida y la muerte, una comprensión personal de la espiritualidad y los conceptos espirituales, y la identificación de las necesidades espirituales del paciente.
4	Reflexiones grupales.^26^	4 sesiones	Irán	Estudiantes de enfermería	Momennasab, M. et al., 2019	La espiritualidad como búsqueda de significado y propósito. Relaciones con Dios, relaciones con los demás y relaciones con el medio ambiente. Perdón, oración, rituales religiosos, dar esperanza y la presencia de familiares y cuidadores.
5	Programa de entrenamiento mindfulnes.^20^	6 sesiones	España	Profesionales de salud	N. Sansó et al., 2018	Fortalecimiento de la espiritualidad a través de la práctica del conocimiento basado en la experiencia. Uso de la respiración y el cuerpo.
6	ASSET (Actioning Spirituality and Spiritual Care Espiritualidad y Atención Espiritual en Enfermería) El modelo tiene tres componentes principales: estructura y Estructura y contenido, proceso y resultado.^28^	1 sesión	USA	Enfermeras	O'Shea, ER et al., 2011	Sugiere una conciencia de la propia espiritualidad y su perspectiva sobre la prestación de atención espiritual. Autoconocimiento, espiritualidad y El lado espiritual del cuidado.
7	Clases y talleres con acompañamiento del docente - trabajo independiente con ejercicios de reflexión y estudio bibliográfico.^21^	4 sesiones	Países Bajos	Enfermeras	JP. Vlasblom et al., 2011	Espiritualidad desde el componente religioso y desde una visión existencial. Experiencias y propósito de vida. Trascendencia - Cultura. Reconocimiento de necesidades espirituales y mejora de la comunicación para la prestación de los cuidados espirituales
8	Aprendizaje autónomo por correspondencia. Libro de trabajo con un DVD complementario.^22^	7 sesiones	USA	Enfermeras	EJ Taylor et al., 2008	Escucha el dolor espiritual, Dar sentido a lo que se escucha en el marco de la atención espiritual. Creación de respuestas verbales a las declaraciones de los pacientes sobre el dolor espiritual.
9	Teoría de Burkhart/ Hogan. Practica reflexiva. Sesiones didácticas en grupo. Foros. Compartir experiencias.^27^	Sesiones trasversales en el plan de estudios	USA	Estudiantes	Burkhart, L. and Schmidt, W., 2012	El concepto de espiritualidad, reflexiones sobre cómo se puede vivir la espiritualidad / Ejercicios de identidad personal del enfermero.
10	Curso de cuidado espiritual. Reflexión y dinámicas de grupo.^23^	10 sesiones	Países Bajos	Estudiantes	van Leeuwen R et al., 2008.	El concepto de espiritualidad en el proceso de enfermería. Habilidades de comunicación en relación a la relación cuidador-paciente (evaluación espiritual y apoyo espiritual) y el cuidado espiritual en el contexto de equipos interdisciplinarios. Reflexiones sobre experiencias personales relacionadas con aspectos del cuidado espiritual en la sociedad en la práctica de enfermería.

**Tabla 4:** Comparación del efecto en algunos de los estudios de intervención en cuidado espiritual

Autor - Año	Intervención	Pre M (SD	Post M (SD)	Control	Pre M (SD)	Post M (SD)
Fernández-Pascual et al. 2020^19^	Spiritual Care as a Specific Nursing Competence. (Los cuidados espirituales como competencia específica de enfermería)	31.28 (4.87)	35.53 (4.35)		Sin control	
	Attention to One’s Own spirituality. (Atención a la propia espiritualidad)					
	Total, Nursing Students’ Spirituality Training Questionnaire (NSSTQ). Formación Espiritual de los Estudiantes de Enfermería Cuestionario (NSSTQ)).					
Vargas-Escobar and Guarnizo-Tole. 2020^24^	Perception of spirituality. (Percepción de la espiritualidad)	68,7(14.9)	78.1(13.6)	Perception of spirituality (Percepción de la espiritualidad)	68.7(11.3)	68.7(18.2)
	Perception of spiritual care. (Percepción de la atención espiritual)	78.5(78.5)	85.7(85.7)	Perception of spiritual care (Percepción de la atención espiritual)	78.5.9(8.6)	83.9(15.5)
	Perception of personalized care. (Percepción de la atención personalizada)	66.6(17.8)	83.3 (14.2)	Perception of personalized care (Percepción de la atención personalizada)	75(12.1)	75 (18.8)
Hu et al. 2019^25^	Spiritual health (Salud espiritual)	90.3 (13.12)	106.5 (8.31)	Spiritual health (Salud espiritual)	91.6(12.05)	95.0(11.91)
	Spiritual care competency. (Competencia en atención espiritual)	79.2 (20.70)	110.8(9.40)	Spiritual care competency (Competencia en atención espiritual)	110.8(9.40)	95.6(15.22)
Momennasab, M. et al. 2019^26^	Total, spiritual well-being. (Bienestar Espiritual)	95.83 (13.36)	102.36 (11.33)	Total, spiritual well-being. (Bienestar Espi-ritual)	95.57(11.43)	95.12 (13.86)
N. Sansó et al. 2018^20^	Nurse' Perspectives TowardProviding Spiritual Care. (Perspectivas de los enfermeros En la atención espiritual)	27.2 (6.45)	21.0(6.91)		Sin control	
JP. Vlasblom et al., 2011^21^	Report about the patient's spiritual questions and/or needs: Until now, no reports are written anywhere yet. (Informe sobre las preguntas y/o necesidades espirituales del paciente)	18	0			
	Report is written by: the nurse. (Informe redactado por: la enfermera)	77	97		Sin control	
	Manner of discovering spiritual questions by nurses: By asking if the patient desires to go to the consultationcentre, silence centre or the chapel. (Forma de descubrir las cuestiones espirituales por parte de los enfermeros: Preguntando al paciente si desea ir a la consulta, al centro de silencio o a la capilla.)	27	52			
	Patients' opinions about nursing care: have experienced sufficient support from the nurses in dealing with my Illness. (Opinión de los pacientes sobre los cuidados de enfermería: he recibido suficiente apoyo de las enfermeras para hacer frente a mi enfermedad.)	69	85	Patients' opinions about nursing care: have experienced sufficient support from the nurses in dealing with my Illness. (Opinión de los pacientes sobre los cuidados de enfermería: he recibido suficiente apoyo de las enfermeras para hacer frente a mi enfermedad.	75	77
EJ Taylor et al. 2008^22^	Spiritual Care Perspective Scale-Revised-SCPS-R	39.2(5.6)	42.2(4.3)		Sin control	
	Daily Spiritual Experience Scale - DSES. (Escala de Perspectiva de Atención Espiritual-Revisada-SCPS-REscala de Experiencia Espiritual Diaria - DSES)	40.8(13.6)	37.6(12.8)			
	Response Empathy Scale RES. (Escala de empatía de respuesta RES)	31.6(7.9)	43.8(8.4)			
	Communicating for Spiritual CareTest - CSCT. (Comunicación para la atención espiritualTest - CSCT)	13.3(3.6)	15.3(3.9)			
after six weeks						
van Leeuwen R et al. 2008^23^	Assessment and implementation of spiritual care. (Evaluación y aplicación de la atención espiritual.)	21.9(3.1)		Assessment and implementation of spiritual care. (Evaluación y apli-cación de la aten-ción espiritual.)		22.0 (3.2)
	Professionalisation and improving quality of spiritual care (Profesionalización y mejora de la calidad de la atención espiritual)	20.4(3.4)		Professionalisation and improving quality of spiritual care. (Profesionaliza-ción y mejora de la calidad de la atención espiritual)		19.4(2.9)
	Attitude towards the patient’s spirituality. (Actitud hacia la espiritualidad del paciente)	16.24 (1.5)		Attitude towards the patient’s spiri-tuality. (Actitud hacia la espiritualidad del paciente)		16.3(1.6)
	Communication. ( Comunicación)	8.4(0.9)		Communication (Comunicación)		8.4(0.7)

## Discusión

La revisión exhaustiva de la literatura identificó evidencia clave sobre el cuidado espiritual y el papel activo de los profesionales de enfermería en la satisfacción de las necesidades espirituales de los pacientes^30^. Por otro lado, se reconoce que los profesionales de la salud ignoran la dimensión espiritual, lo que amenaza el abordaje holístico del cuidado^31^; si se considera la complejidad de los conceptos de espiritualidad y cuidado espiritual en los estudios revisados, se propusieron varios enfoques pedagógicos innovadores, que fueron implementados en varios proyectos de cursos de pregrado y posgrado para enfermeros y enfermeras de diferentes países y que demostraron un resultado positivo de las intervenciones.^19^^-^^24^^,^^26^^-^^29^. También es importante mencionar los estudios descritos en la literatura que, aunque no hayan propuesto métodos experimentales, han formulado temas para fortalecer la capacidad de atención espiritual a través de enfoques educativos integrados en las dimensiones teórica y clínica basados algunos en la teoría de Benner.^30^

Los estudios de intervención en la educación del cuidado espiritual han mostrado cambios significativos en las habilidades de formación de los estudiantes y profesionales de enfermería, tales como estrategias de comunicación, evaluación y documentación de las necesidades espirituales de los pacientes y formulación de planes de atención de enfermería que se enfocan en cuidar a los pacientes y sus familias de manera integral. También en los resultados del análisis, las enfermeras informaron que se sentían mejor preparadas para los problemas espirituales y podían discutir estos problemas con los pacientes.^19^^-^^24^^,^^26^^-^^29^ Seria de interés en futuros estudios poder analizar si estos resultados tienen relación con el desempeño académico de los y las estudiantes de los programas de enfermería.

Por otra parte, los contextos en los que se desarrollaron las intervenciones van desde ambientes netamente educativos, hasta escenarios de práctica clínica formativa; en cuanto al número de sesiones varían desde una sesión hasta sesiones de tipo trasversal a través del currículo^24^^,^^27^. También se realizaron intervenciones se realizaron usando espacios de atención de pacientes en igual proporción^21^^,^^22^^,^^27^^,^^28^_._

Una limitante del estudio es los pocos estudios de intervención realizados con grupo control y aleatorización lo que puede presentar una debilidad en los efectos presentados; al respecto se requieren mayores estudios que midan el efecto de intervención con grupo control. Otra falencia es la poca claridad que presentan los estudios respecto a las estrategias pedagógicas presentadas, donde se presentan más las didácticas y formas en que se desarrollaron las sesiones educativas, sin declarar un modelo pedagógico claro que respalde conceptual y epistemológicamente la estrategia educativa utilizada.

Otros estudios realizados en Europa^3^^,^^6^ pero no incluidos en el estudio han permitido la consolidación de la red EPICC (Improving Nursing to Provide Spiritual Care through Innovative Education and Compassion) Nursing Competence) que es fruto del proyecto Erasmus con la Unión Europea, alli se relaciona con la necesidad de que las enfermeras aborden las creencias personales, religiosas y espirituales de los pacientes como parte de los cuidados integrados centrados en la persona^32^. El punto de partida para el desarrollo de este proyecto fue la necesidad de comprender cómo los estudiantes de enfermería adquieren competencia en la atención espiritual y cómo la educación en enfermería y partería en Europa aborda este tema. Es por ello que, en la educación de los estudiantes sobre el cuidado espiritual, es importante comprender las actitudes de los estudiantes frente al tema ^33^, así como tener en cuenta su propia cultura para definir si los entornos académicos y clínicos en el hospital son propicios para el aprendizaje.

## Conclusión

Existe evidencia suficiente sobre el abordaje de la espiritualidad personal como punto de inflexión en el desarrollo de la competencia de cuidado espiritual en profesionales y estudiantes de enfermería.

El concepto de espiritualidad intrapersonal, incluida la comprensión de la espiritualidad propia, la espiritualidad interpersonal, la trascendencia y el significado de la vida, se identificaron como temas centrales en los planes de estudio enfocados a mejorar la competencia en cuidado espiritual de los estudiantes y profesionales de enfermería.

Las recomendaciones para las intervenciones educativas para lograr un aprendizaje del cuidado espiritual incluyeron: el aprendizaje experiencial, la práctica reflexiva, juego de roles y la escritura narrativa, también el uso del arte para expresar conceptos complejos de cuidado espiritual, los debates sobre el cuidado holístico, y la participación en investigaciones sobre el tema.
